# Success rate of the treatment of early childhood caries under general anesthesia: A retrospective cohort study in different periods

**DOI:** 10.3389/fped.2023.1117935

**Published:** 2023-03-31

**Authors:** Baize Zhang, Junhui Wang, Xinxin Han, Ruohao Fang, Zirui Wang, Zeming Hui, Yujiang Chen, Jiajia Liu, Xiaojing Wang

**Affiliations:** ^1^ Department of Pediatric Dentistry, School of Stomatology, Fourth Military Medical University, Xi’an, China; ^2^ Chinese PLA 94498 Army Hospital, Nanyang, China; ^3^ Health Team, Naval Aviation Unit 92497, Southern Theater Command, Lingshui, China; ^4^ Chinese PLA 94162 Army Hospital, Xi'an, China

**Keywords:** early childhood caries, general anesthesia, preformed crown restoration, the three-year success rate, treatment

## Abstract

**Instruction:**

The purpose of this study was to evaluate the three-year success rate of the treatment for early childhood caries (ECC) under general anesthesia in different periods (2011 and 2018).

**Methods:**

Children (<6 years old) who had severe caries and were treated under general anesthesia in 2011 and 2018 were selected and followed up by telephone appointment and clinical examination. Success rate of each treatment was determined and possible factors associated with treatment failure were evaluated.

**Results:**

There were 153 patients (with an average age of 48.55 ± 13.37 months) and a total of 2,018 teeth included in the 2011 group. In the 2018 group, there were 273 patients with an average age of 49.01 ± 12.42 months and a total of 3,796 teeth. The success rate in the 2011 group was significantly lower than that in the 2018 group. Teeth with mineral trioxide aggregate (MTA)-capped pulp survived significantly longer than those with calcium hydroxide-capped pulp. The utilization rate of preformed crown restoration was higher than that of resin restoration, and the survival time of dental restorations with preformed crown was prolonged. For posterior teeth, the success rate of indirect pulp capping and pulpotomy was also significantly higher than those without preformed crowns.

**Discussion:**

General anesthesia is a safe and effective behavioral management method for uncooperative children's dental treatment. The use of biocompatible pulp capping materials and preformed crowns improved the success rate of treatment and prolonged the survival time of affected teeth.

## Introduction

Early Childhood Caries (ECC) is one of the most common chronic oral diseases with a high incidence and serious harm to children's oral health, especially in developing countries (1). The 3rd National Oral Health Survey in the Mainland of China conducted in 2005 showed that the prevalence of dental caries in 5-year-old children was 66%, the Decayed, Missing and Filled Teeth (dmft) index was 3.5, and about 97% of caries were untreated. The 4th National Oral Health Survey in the Mainland of China conducted in 2015 showed that the prevalence of dental caries in children aged 3, 4, and 5 was 50.8%, 63.6%, and 70.9%, respectively, and the average dmft index was 2.28, 3.40 and 4.24, respectively (2). The incidence of caries in 2015 was increased comparing to that in 2005. Caries typically occurred early in young children with extensive damages and rapid progresses. Most of the children cannot cooperate with the treatment due to their young age. Studies have shown that about 20.1% of children aged 4 to 6 years showed non-cooperation in oral diagnosis and treatment (3). The therapeutic efficacy under restraint condition cannot be guaranteed and has an impact on the physical and mental development of children. How to effectively deal with young patients who cannot cooperate with treatment for caries is a challenge faced by pediatric dentists.

Dental general anesthesia (DGA) can eliminate the interference of uncooperative behaviors, e.g., crying and struggling during treatment, provide high-quality dental treatment in a safe environment, reduce anxiety during follow-up visits and improve cooperation (4). It provides a safe and effective behavioral management method for young children with multiple decayed teeth or dental anxiety. As a developing country, China started to carry out oral treatment for children under general anesthesia in 1999 (5). At the beginning, many people questioned the necessity of general anesthesia because of its high risk, high cost, and high failure rate. With the advancement of children's oral treatment techniques, and the society's attention on children's physical and mental health, treatment under general anesthesia has been increasingly recognized by parents. At present, there is no comparative studies on the success rate of treatment under general anesthesia in the earlier and more recent period in Northwest China.

In this study, we followed up children treated under general anesthesia in earlier and more recent period, and evaluated the success rate of different treatments for various oral diseases (6). We expect to provide references for medical institutions undertaking oral treatments under general anesthesia.

## Materials and methods

This is a retrospective study approved by the Medical Ethics Committee of the Stomatological Hospital of Fourth Military Medical University (IRB-REV-2015020). This study included 153 subjects in 2011 and 273 subjects in 2018 with at least 3 follow-up records for each subject. The inclusion criteria were as follows: (a) children under the age of 6 years treated for caries; (b) children with anesthesiology category I; and (c) all treatments performed by the same three doctors and with at least 3 follow-ups in the medical records. The exclusion criteria were as follows: (d) children treated under general anesthesia for other reasons, e.g., dental trauma, supernumerary teeth, and frenulum trimming; and (e) Children with systemic diseases, e.g., autism and cerebral palsy. Informed consent from the guardians was obtained for this study.

## Data collection

**Figure 1:**
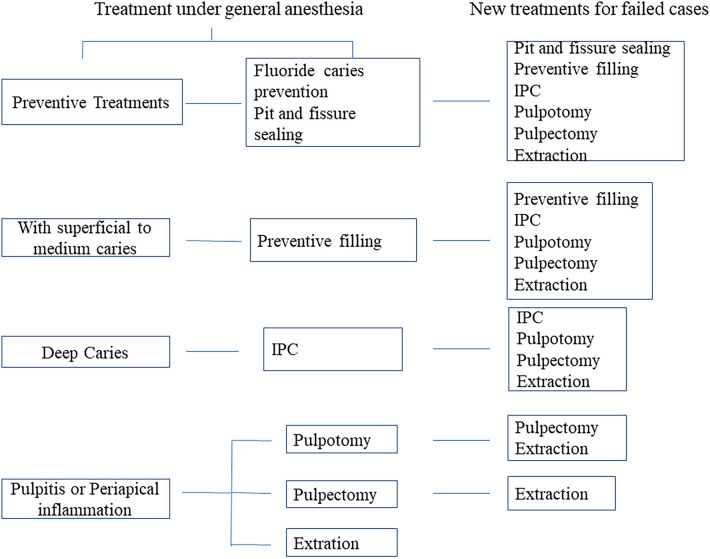
Treatment of different diseases under general anesthesia and treatment after failure.

The study included records of clinical examinations before surgery and all the follow-up records for 3 years following general anesthesia will be recorded. All preoperative examinations and treatments in this study were performed by three dentists who had experience in pediatric oral care for more than 5 years and all children received the same treatment procedures in the management of early childhood caries (ECC) (Figure 1). Follow-up examinations were performed by two additional trained examiners blinded to the treatment. Consistency test was performed for the two examiners with a Kappa value of 0.85. When assessment results differed among the two examiners, additional examinations were performed after two weeks. The date of follow-up for each patient was also recorded. All dental conditions including additional treatment were recorded at each follow-up visit. Failure of the treatment was considered if the following conditions occur (7). Treatment of pit and fissure sealing was considered to have failed if part of the sealant was lost, or caries (including secondary caries and new caries) were formed in the same tooth. Restoration or preformed crowns were considered to have failed if (a) the edges of the restoration were unfit, loose, fractured, perforated, or falling off; (b) the affected tooth has secondary caries or recurrent caries; and (c) endodontic symptoms appeared in patients who did not receive endodontic treatment. Endodontic treatments (including indirect endodontic treatment, pulpotomy, pulpectomy, and root canal treatment) were considered to have failed if (a) the patients were sensitive to percussion; (b) there were local pains; (c) there was presence of swelling or abscess; and (d) there was radiological evidence of pathological shadows in the periapical site or root furcation. Treatment with gap retainer was considered to have failed if the retainer fell off or broke.

In addition, if the child had a problem with the tooth before the follow-up time and was treated in the emergency room, then the treatment was also judged to have failed. In the follow up period, if the tooth needs to be removed due to secondary inflammation, the treatment was considered to have failed. If the extraction of the affected tooth was due to the normal eruption of the inherited permanent tooth and the retention of the deciduous tooth, the treatment was not considered to be a failure.

## Statistical analysis

Data entry and statistics were performed on patients or affected teeth. Descriptive analysis was expressed by frequency and success rate. The success rate was compared among different treatments by Chi-square and Fisher's exact tests. Cox regression was used to analyze the factors (age, gender) that influence the risk of treatment failure. Kaplan-Meier method was used to analyze the survival curve of different treatments. Wilcoxon test was used for the comparison of two different curves. SPSS (20.0) was used for all data collection and analysis, and the test level was *α* = 0.05.

## Results

### General information

A total of 177 cases were included in the study in the 2011 group. During the 3-year follow-up, 24 cases were lost to follow-up, with a loss rate of 15.7%. A total of 294 cases were included in the 2018 group. During the 3-year follow-up, 273 cases were returned to the clinic on time, and the lost follow-up rate was 7.7%.

Table 1 showed the general information of the children included in this study. In 2011, there were 153 cases (male: 76, female: 77, including 2,018 teeth and an average of 13.90 ± 3.27/patient), and the age at the time of treatment was 2–6 years old with an average age of 4.05 ± 1.11 years. In 2018, there were 273 cases (male: 144, female: 129, including 3,796 teeth and average of 13.90 ± 3.27 teeth/patient) The youngest child at the time of treatment was 1.67 years old. The teeth involved in this study were all primary teeth.

**Table 1:** General information of the patients included in this study.

Variables	2011	2018	t/*χ*^2^	*P*
Gender, *n* (%)			0.371	0.542
Male	76 (49.7)	144 (52.75)		
Female	77 (50.3)	129 (47.25)		
Age, *n* (%)			1.349	0.718
<3	25 (16.34)	43 (15.76)		
3	60 (39.21)	102 (37.36)		
4	35 (22.88)	76 (27.84)		
>5	33 (21.57)	52 (19.05)		
Average age	48.55 ± 13.37 m	49.11 ± 12.39m	−1.940	0.052
**Number of teeth**				
Total	2018	3796	–	–
Average	13.19 ± 3.44	13.90 ± 3.27	−485.94	<0.001
Distribution of the tooth number, *n* (%)			3.107	0.375
0–5 teeth	3 (1.96)	2 (0.73)		
6–10 teeth	26 (17.00)	37 (13.55)		
11–15 teeth	90 (58.82)	159 (58.25)		
16–20 teeth	34 (22.22)	75 (27.47)		
Position of the teeth, *n* (%)			4.006	0.549
Anterior teeth	950 (47.08)	1,705 (44.92)		
Posterior teeth	1,068 (52.92)	2,091 (55.08)		
The first primary molar	556 (52.06)	1,058 (50.60)		
The second primary molar	512 (47.94)	1,033 (49.40)		
Maxilla	1,242 (61.55)	2,345 (61.78)		
Mandible	776 (38.45)	1,451 (38.22)		

### Success rate of pit and fissure sealing under general anesthesia

In the 2011 group, the preventive treatment rate for the posterior teeth without caries was 0. In 2018, pits and fissures sealing were performed for 86 teeth, accounting for 43.65% of the posterior teeth without caries (86/197). The three-year success rate was 63.77%, which was significantly different from the teeth without caries. There was no significant difference in the three-year success rate of pit and fissure sealing between the first and second primary molars. The main reason for the treatment failure was the development of new caries on the adjacent surface (44/69). For the new caries, restorations with preformed crowns were performed during the follow-up examinations.

### Pulpal treatments (indirect pulp capping, pulpotomies,and pulpectomies)

During the three-year follow-up period, the overall success rates of indirect pulp capping (IPC), pulpotomy, and pulpectomy in the 2011 group were 53.63%, 47.37%, and 58.87%, respectively. The success rate of indirect pulp capping for the anterior teeth and posterior teeth in the 2011 group was 54.35% (50/92) and 53.21% (83/156), respectively. The success rate of indirect pulp capping for the anterior teeth and posterior teeth in the 2018 group was 84.95% (525/618) and 81.47% (561/678), respectively. The success rate for the anterior teeth was higher than that for the posterior teeth. There was no significant difference between the success rate for the anterior teeth and that for the posterior teeth in the same period. However, the success rate for the anterior teeth in the 2018 group was significantly different from that in the 2011 group (54.35% VS 84.95%, *P *= 0.03). The success rate for the posterior teeth in the 2018 group was also significantly different from that in the 2011 group (53.21% VS 81.47%, *P *< 0.001). During the same period, the success rates for the first and second primary molars were not significantly different (52.70% VS 53.66% in 2011, 83.23% VS 82.34% in 2018). However, the success rate was significantly different between the 2011 and 2018 groups (52.70% VS 83.23% for the first molar, 53.66% VS 82.34% for the second molar) (*P *< 0.001). For indirect pulp capping, the risk of failure in 2011 was 1.7 times higher than that in 2018 (hazard ratio [HR] equals 1.77; 95 percent confidence interval [CI] equals 1.499 to 2.081; *P *< 0.001). In terms of the selection of pulp capping agent, the utilization rate of calcium hydroxide in the 2011 group and 2018 group was 92.52% and 51.78%, respectively. The utilization rate of MTA (or iRoot BP) in 2011 group and 2018 group was 7.48% and 48.22%, respectively (*P *< 0.001).

In primary teeth with extensive caries but without apical lesions, pulpotomy can preserve the root pulp tissues when pulp exposure occurs due to caries or mechanical reasons and facilitate physiological replacement of primary teeth with permanent teeth (8). The success rates of pulpotomy for the anterior teeth in 2011 group and 2018 group were 43.47% (10/23) and 70.37% (19/27), respectively (*P *= 0.06). The success rates of pulpotomy for the posterior teeth in the 2011 group and 2018 group were 50.00% (17/34) and 85.90% (67/78), respectively (*P *< 0.001). In the 2011 group, the success rate for the first primary molars was higher (but not significantly) than that for the second primary molars (60.00% VS 35.71%, 12/20 VS 5/14, *P *= 0.30). The opposite results were observed in the 2018 group (81.82% VS 91.18%, 36/44 VS 31/34, *P *= 0.078). The success rates for the first primary molars in the 2011 group and 2018 group were 60.00% and 81.82%, respectively (*P *< 0.001). The success rates for the second primary molars in the 2011 group and 2018 group were 35.71% and 91.18%, respectively (*P *< 0.001). The risk of pulpotomy failure in the 2011 group was 1.6 times higher than that in the 2018 group (hazard ratio [HR] equals 1.67; 95 percent confidence interval [CI] equals 1.086 to 2.565; *P *< 0.001). With regard to the pulp capping agent, the utilization rates of calcium hydroxide in the 2011 group and the 2018 group were 81.65% and 0 respectively. The utilization rates of MTA (or iRoot BP) in the 2011 group and the 2018 group were 18.35% and 100.00%, respectively (*P *< 0.05).

**Figure 2:**
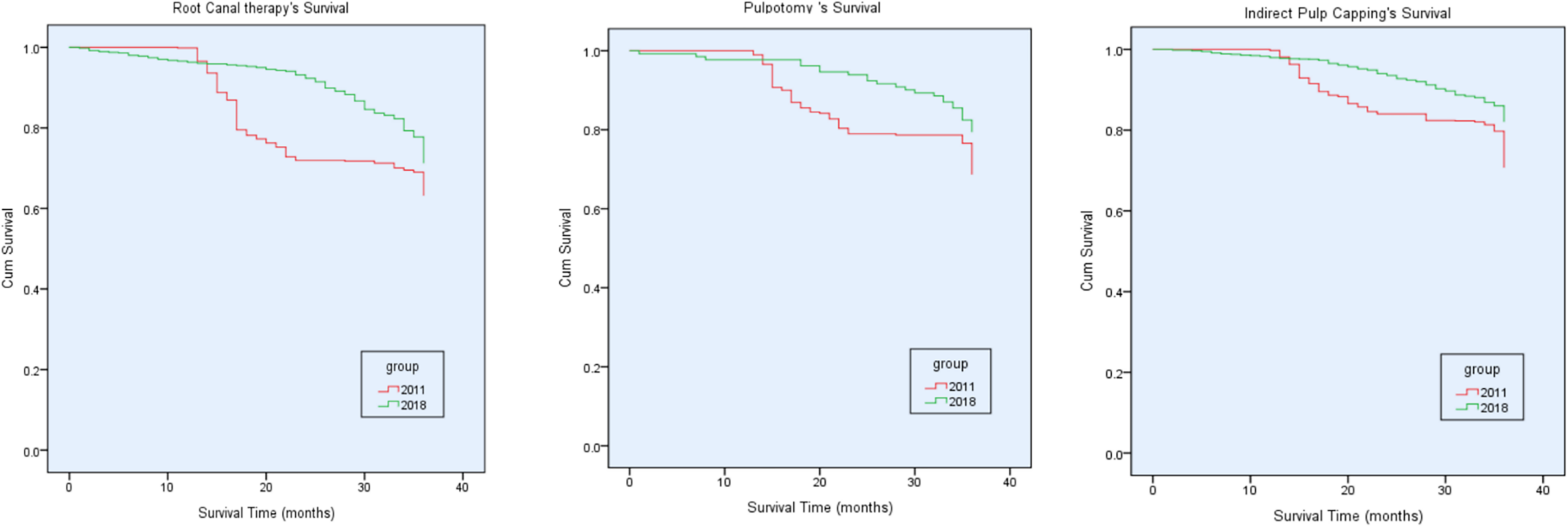
Kaplan-Meier survival curves of teeth with indirect endodontic treatment, pulpotomy and pulpectomy between the 2011 group and the 2018 group.

Pulpectomy is used to treat teeth with irreversible infection or necrosis of pulp tissues due to caries or trauma. In the 2011 group, there was no significant difference in the success rate of pulpectomy between the anterior and posterior teeth, or between the first and second primary molars. The success rate for the anterior teeth, the posterior teeth, the first primary molar, and the second primary molar was 63.41%, 50.00% (21/42), 46.15% (12/26), and 56.25% (9/16), respectively. In the 2018 group, there were significant differences in the success rate of pulpectomy between the anterior teeth and posterior teeth, or between the first and second primary molars. The success rate for the anterior teeth, the posterior teeth, the first primary molar, and the second primary molar was 61.60% (308/500), 78.15% (296/379), 85.58% (267/312) and 85.58% (267/312), respectively. There was significant difference in the success rate of pulpectomy for the posterior teeth, the first primary molars, or the second primary molars between 2011 group and 2018 group. The risk of pulpectomy failure in 2011 was 1.4 times higher than that in 2018 (hazard ratio [HR] equals 1.6; 95 percent confidence interval [CI] equals 1.24 to 1.72; *P *< 0.001). Table 2 showed the comparison of the success rates of each treatment at different follow-up period. Figure 2 showed the comparison of Kaplan-Meier survival curves teeth with indirect endodontic treatment, pulpotomy and pulpectomy between the 2011 group and the 2018 group.

**Table 2:** Comparison of the success rates of each treatment at different follow-up times in 2011 and 2018.

Treatment		0–12 month			13–24 month			≥25 month		
		Number of teeth with successful treatment	Number of teeth with failed treatment	*P*	Number of teeth with successful treatment	Number of teeth with failed treatment	*P*	Number of teeth with successful treatment	Number of teeth with failed treatment	*P*
Filling	2011	233	0	0.006	209	24	0.109	67	28	0.001
	2018	50	3		49	1		42	2	
Pulp capping	2011	862	2	<0.001	717	136	<0.001	133	115	<0.001
	2018	1792	37		1698	81		1086	210	
Root canal treatment	2011	580	1	<0.001	418	162	<0.001	73	51	0.001
	2018	1462	56		1391	61		871	320	
Extraction	2011	50	0	–	49	0	–	13	0	–
	2018	179	0		178	0		134	0	
Pulpotomy	2011	290	0	0.03	219	61	<0.001	27	30	<0.001
	2018	128	3		122	5		86	19	
Pit and fissure sealing	2011	0	0	–	0	0	–	0	0	–
	2018	86	0		72	14		44	25	

### Restorative treatment

Restorations after various treatments include glass-ionomer restorations, resin restorations and preformed crown restorations (including anterior transparent strip crown restorations and posterior metal preformed crown restorations). In the treatment of posterior teeth restoration, 721 teeth in the 2011 group used resin restorations, accounting for 67.51%, and glass-ionomer restorations accounted for 2.25%. In the 2018 group, 510 teeth were repaired by resin, accounting for 24.39%, while only 0.62% were repaired by glass-ionomer. The results of follow-up showed that 287 teeth (38.52%) had secondary or secondary caries after resin and glass-ionomer restorations in the 2011 group, and 98 teeth (18.74%) had secondary or secondary caries after glass-ionomer and resin restorations in the 2018 group.

In the 2011 group, 55 of the 950 (5.79%) anterior teeth were treated with transparent strip crowns, and 762 of the 1,705 (44.69%) anterior teeth were treated with transparent strip crowns in the 2018 group. In the 2011 group 323 of 1,068 (30.24%) posterior teeth were treated with metal preformed crowns and in the 2018 group 1,568 of 2,091 (30.24%) posterior teeth were treated with metal preformed crowns. In the follow-up examinations, perforation and falling off of metal preformed crowns occurred in 5.54% and 4.89% of the patients in the 2011 group the 2018 group, respectively (*P *> 0.05). Table 3 showed the use of preformed crowns after each treatment in the two groups. Table 4 showed the difference in success rate between the specific treatment methods, and whether anterior transparent resin crowns and posterior metal preformed crowns were or were not used. In the indirect pulp capping and pulpotomy of posterior teeth, the success rate of using a preformed crown was significantly higher than that without using preformed crown (*P *< 0.05). In the pulpectomy, the success rate of using a preformed crown was not significantly different from that without using preformed crown.

**Table 3:** Comparison of the utilization rate of preformed crowns after different treatments in 2011 and 2018.

Treatments		Anterior teeth (%)	Posterior teeth (%)	Utilization rate (%)
Filling	2011	3.42	5.17	4.29
	2018	43.09	66.77	52.83
Pulp capping	2011	4.59	13.77	9.84
	2018	43.09	66.77	55.44
Root canal treatment	2011	7.16	69.92	33.73
	2018	52.32	97.36	78.19
Pulpotomy	2011	11.47	37.93	30.00
	2018	65.79	81.72	77.10

Note: *χ*^2 ^= 15.362, *P *= 0.002.

**Table 4:** Comparison of success rates of preformed crown restoration after different treatments in 2011 and 2018.

Treatment	Year	2011			2018		
		Number of teeth with successful treatment	Number of teeth with failed treatment	*P*	Number of teeth with successful treatment	Number of teeth with failed treatment	*P*
**Pulp capping**							
Posterior teeth	With preformed crown restoration	63	5	1.000	588	49	<0.0001
	Without preformed crown restoration	270	156		189	128	
**Root canal treatment**							
Posterior teeth	With preformed crown restoration	146	26	<0.0001	665	184	0.799
	Without preformed crown restoration	31	43		19	4	
**Pulpotomy**							
Posterior teeth	With preformed crown restoration	66	11	<0.0001	68	8	0.001
	Without preformed crown restoration	77	49		9	8	

## Discussion

The prevention and treatment of uncooperative children's dental disease has long been a challenge and difficulty in oral clinical work. Studies have shown that psychological behavior induction and compulsory restraint measures can be used for children who are young, uncooperative and have fewer teeth. However, the subjective fear and anxiety levels will increase after such treatment (4), increasing the difficulty of subsequent treatments. General anesthesia provides an effective and safe behavioral management measure for the treatment of dental disease in uncooperative children. General anesthesia was first used in the United States for the treatment of children's dental disease in 1951 (9), and has become a routine auxiliary means for children's oral treatment in Europe and the United States. DGA was introduced in Northwest China in 2009, but it was not well recognized and accepted by parents. Even if dentists provide sufficient explanation on the indications of DGA, it is still difficult for parents to accept it. Therefore, at the beginning, there were relatively few cases treated under general anesthesia. At the same time, because the medical development in the Northwest region was relatively slow, many children's oral treatment techniques and materials have not been renewed in time and the therapeutic effect has not reached an ideal goal. With the development of science and technology and the improvement of restorative materials, general anesthesia was increasingly accepted, and the number of patients treated under anesthesia has been increasing. The Stomatological Hospital of the Air Force Military Medical University is the largest dental specialty hospital in Northwest China. Since general anesthesia was introduced in 2009, the number of cases was less than 80 per year before 2011, and after 2015, there were more than 400 cases treated per year, and about 200 patients were still waiting for the treatment each year due to limited medical resources.

The purpose of this study was to evaluate the three-year success rate of teeth treated under general anesthesia in the early and more recent period after DGA was introduced, and to provide evidence for dentists and parents to choose appropriate procedures during clinical treatment. Among 177 patients in the 2011 group, 24 cases were lost to follow-up, with a loss rate of 15.7%. However, with the development of technology and the improvement of system, only 21 cases were lost to follow-up among 294 patients in the 2018 group, with a loss rate of 7.7%. The loss of follow-up rate of both groups was low, which further indicated that the included patients had good oral hygiene compliance, and the results of the study were more credible. In the early period (the 2011 group), treatments failed in 41.71% of the teeth after three years of follow-up, while treatments failed in 20.29% of the teeth in more recent period (the 2018 group). Overall, the same treatment for the same disease by the same doctor also had a higher success rate in 2018 than in the 2011 group. We speculate that the reasons for the reduced failure rate in 2018 could be due to the advancement in treatment techniques over time, increases in dentists' experiences, and the high performance materials.

Parents usually assume that DGA can solve all the dental health problems of the children, and the short-term oral health status after DGA do strengthen this assumption. However, the occurrence and development of caries has its own uniqueness, and it is difficult to eliminate all dental problems with one treatment. Therefore, it is of great clinical significance to evaluate the success rate of various treatments and to explore measures to improve the success rate. The filling materials used in this study included glass-ionomer, resin materials and metal preformed crowns. Glass-ionomer was used as filling material to bond with tooth tissue by its chemical properties. However, due to its hydrophilic properties, it is easy to absorb water and dissolve after filling, thus reducing its success rate (10). In this study, the 3-year failure rate of composite resin as a filling material was 38.52% in the 2011 group, which was basically consistent with the 30-month survival rate of 34.8% in the Pamela Campagna study of composite resin for severe infantile caries (11). The reason is that some of the dental fills of primary molars after resin restoration under general anesthesia fell off during the three years of follow-up. Secondary caries, and new caries occurred in some teeth or in adjacent surfaces, which is consistent with previous randomized clinical trials (12). A number of studies have shown that the application of metal preformed crowns has significantly improved the success rate of treatment with a success rate ranging from 90% to 100% (13–16), which was also verified by the results of this study. The preformed crown has a protective effect on the teeth after treatment, avoids the occurrence of secondary caries and new caries, and greatly improves the success rate of the treatment. Therefore, the long-term efficacy of metal preformed crowns for posterior teeth is superior to that of the resin composites.

There are few reports of clinical trials on the pit and fissure sealing of primary molars. Some studies have shown that the retention rate of pit and fissure sealant after 1 year is 82%–88.6%, and the retention rate is 74% after 2.8 years (14, 15). Due to the combined effect of anatomical shape, tissue structure, mineralization degree of primary teeth, the probability of caries in pit and fissure is about 9 times higher than that in the smooth surfaces (16). Therefore, pit and fissure sealing is one of the main measures to prevent caries in primary teeth. In the early period, parents and dentists focus more on solving the pain of children and focus less on the prevention. Therefore, the utilization rate of pit and fissure sealing in the 2011 group was 0. In the 2018 group, pit and fissure sealing was conducted in 43.65% of the teeth without caries, and the rate of pit and fissure sealant falling off was 0 in the first year after treatment. The failure rate at the third year was 36.33%, which was higher than that reported in the literature, but was significantly different from those without caries. Possible reasons for the high failure rate were the underestimated success rate. This is because resin restoration or crown restoration due to the caries on the adjacent surface should not be considered as pit and fissure sealant applied on the occlusal surface. This also confirms that pit and fissure sealing is an effective measure to prevent caries on the occlusal surface, but its long-term clinical efficacy needs to be verified by more randomized trials. Our results are consistent with many studies showing that indirect endodontic treatment and pulpotomy have a high success rate (12, 17). The success rate in the 2018 group is higher than that in the 2011 group, which is possibly due to the improvement of pulp capping materials and the changes of the restoration methods. Capping materials with good biocompatibility cannot be widely used due to the high cost although they have many advantages. First, in pulpotomy for posterior teeth, the pulp capping materials in the 2011 group included calcium hydroxide (88.54%) and MTA (or iRoot BP) (11.46%), and in the 2018 group, the pulp capping materials included calcium hydroxide (34.54%) and MTA (or iRoot BP) (65.46%). The success rate of pulpotomy using calcium hydroxide varies from 46.1% to 87.5% (13, 18), and the success rate of pulpotomy using MTA can reach up to 97% (14, 15). Second, metal preformed crowns are widely used recently. In the 2011 group, metal preformed crowns were less used, and it was performed only in 13.77% of pulp capping procedure and 37.93% of pulpotomy. After resin restoration, there are more pulp inflammations caused by secondary caries and loss of fillings, and thus failure rate increases. In the 2018 group, the utilization rate of preformed crowns after various treatments increased, and the success rate was also significantly improved. However, there was a decrease of the success rate in the third year of the follow-up, which could be due to the decrease in the number of affected teeth and the change in the restoration method in the third year of the follow-up.

The success rate of pulpectomy was relatively low in most studies, and this was consistent with our results in the 2011 group (19). However, the success rate in the 2018 group was relatively high, which could be associated with the patients in the follow-up. Although the success rate of pulpectomy is low, it can avoid tooth extraction, remove infected pulp, and keep the affected tooth as much as possible to maintain its physiological space. This study also showed that the success rate of pulpectomy for the second primary molars was higher than that for the first primary molars, which could be due to the more complicated root canal system of first primary molars.

While discussing the success rate of various treatments, we must consider the strengths and limitations of the study. This study was conducted in the most authoritative stomatology hospital in Northwest China, which is the first medical institution to carry out DGA. The level of its medical technology and clinicians is in line with international treatment guidelines, which reduces treatment bias. The subjects included in this study was highly representative of the region, and the results provide a holistic view of tooth longevity after different treatments. This helps dentists in other medical institutions develop treatment plans and select restorative materials when performing comprehensive pediatric oral care under general anesthesia. However, our study also has its limitations. First, the success rate is mainly based on clinical examination. If periapical inflammation is found in clinical examination, x-ray examination is performed to confirm the diagnosis. Therefore, not all affected teeth should undergo imaging examination. But the occurrence of secondary caries could not be detected in time in clinical examination, which may overestimate the success rate of resin restorations. In addition, the patients treated under general anesthesia come from eight different provinces and cities, and therefore some of the patients could not return for follow-up in time, resulting in the biased results. Third, although this is a comparative study and has a longer follow-up period than most other studies, the specific reasons for the treatment failure were not explored. Fourth, the severity of decay (including the number of tooth surfaces and the location of decay) that affected the outcome was not recorded in this study.

## Conclusions

1. Compared with resin restoration, metal preformed crowns restoration has a higher success rate, which is conducive to improving the success rate of children's dental treatment under general anesthesia.
2. The success rate of indirect endodontic treatment and pulpotomy has been greatly improved with the improvement of restoration materials and techniques and the applicability of these materials to primary teeth.
3. Pit and fissure sealing is an effective measure to prevent dental caries on the posterior occlusal surface.

## Data Availability

The original contributions presented in the study are included in the article/Supplementary Material, further inquiries can be directed to the corresponding author/s.
